# Consumption of Cashew (*Anacardium occidentale* L.) Nuts Counteracts Oxidative Stress and Tissue Inflammation in Mild Hyperhomocysteinemia in Rats

**DOI:** 10.3390/nu14071474

**Published:** 2022-04-01

**Authors:** Ramona D’Amico, Marika Cordaro, Roberta Fusco, Alessio Filippo Peritore, Tiziana Genovese, Enrico Gugliandolo, Rosalia Crupi, Giuseppina Mandalari, Daniela Caccamo, Salvatore Cuzzocrea, Rosanna Di Paola, Rosalba Siracusa, Daniela Impellizzeri

**Affiliations:** 1 Department of Chemical, Biological, Pharmaceutical and Environmental Sciences, University of Messina, Via F. Stagno D’Alcontres 31, 98166 Messina, Italy; rdamico@unime.it (R.D.); aperitore@unime.it (A.F.P.); tgenovese@unime.it (T.G.); gmandalari@unime.it (G.M.); rsiracusa@unime.it (R.S.); dimpellizzeri@unime.it (D.I.); 2 Department of Biomedical, Dental and Morphological and Functional Imaging, University of Messina, Via Consolare Valeria, 98125 Messina, Italy; cordarom@unime.it (M.C.); daniela.caccamo@unime.it (D.C.); 3 Department of Clinical and Experimental Medicine, University of Messina, 98125 Messina, Italy; rfusco@unime.it; 4 Department of Veterinary Sciences, University of Messina, 98168 Messina, Italy; egugliandolo@unime.it (E.G.); rcrupi@unime.it (R.C.); 5 Department of Pharmacological and Physiological Science, Saint Louis University School of Medicine, 1402 South Grand Blvd, St. Louis, MO 63104, USA

**Keywords:** oxidative stress, hyperhomocysteinemia, cashew nut, NRF-2, NF-κB

## Abstract

Hyperhomocysteinemia (HHcy) is a methionine metabolism problem that causes a variety of inflammatory illnesses. Oxidative stress is among the processes thought to be involved in the pathophysiology of the damage produced by HHcy. HHcy is likely to involve the dysfunction of several organs, such as the kidney, liver, or gut, which are currently poorly understood. Nuts are regarded as an important part of a balanced diet since they include protein, good fatty acids, and critical nutrients. The aim of this work was to evaluate the anti-inflammatory and antioxidant effects of cashew nuts in HHcy induced by oral methionine administration for 30 days, and to examine the possible pathways involved. In HHcy rats, cashew nuts (100 mg/kg orally, daily) were able to counteract clinical biochemical changes, oxidative and nitrosative stress, reduced antioxidant enzyme levels, lipid peroxidation, proinflammatory cytokine release, histological tissue injuries, and apoptosis in the kidney, colon, and liver, possibly by the modulation of the antioxidant nuclear factor erythroid 2–related factor 2 NRF-2 and inflammatory nuclear factor NF-kB pathways. Thus, the results suggest that the consumption of cashew nuts may be beneficial for the treatment of inflammatory conditions associated with HHcy.

## 1. Introduction

Homocysteine (Hcy) is a sulfhydryl-containing amino acid that is produced when methionine is demethylated. Hcy can be converted to cysteine via the sulphuration pathway, or remethylated utilizing methyltetrahydrofolate or betaine [1]. 

The clinical definition of hyperhomocysteinemia (HHcy) is a total plasma Hcy above 15 μmol/L. Mild HHcy is induced by a diet lacking in Hcy-lowering vitamins, such as folate, vitamin B6, and/or vitamin B12 [1]. Experiments on induced HHcy (for example, in a rat model induced by methionine dietary overload [2] or by oral L-methionine or subcutaneous DL-Hcy administration [3]) allow for more research into the links between HHcy and other inflammatory illnesses, as well as the processes that underpin these connections. 

Oxidative stress is among the processes thought to be involved in the pathophysiology of the damage produced by HHcy [4]. The main consequence of reactive oxygen species (ROS)-induced oxidative stress is to trigger inflammatory responses, which are mediated mostly by nuclear factor NF-kB. Some researchers believe that Hcy causes atherosclerosis, either by directly harming the endothelium or by modifying the oxidative state of the endothelium. The formation of ROS such as hydrogen peroxide, superoxide anions, and hydroxyl radicals contributes to endothelial Hcy-mediated cytotoxicity [5] during the autooxidation of Hcy to homocysteine or other mixed disulfides [1,6].

Data have revealed that HHcy may be linked to disorders affecting other organs [7]. The influence of HHcy on organs’ oxidative state has been examined in diverse tissues such as the endothelium [8], liver [9], heart, and brain [10,11]. Hcy may also have pathogenetic implications in inflammatory bowel disease (IBD), demonstrating that it is a pro-inflammatory and immunostimulating molecule [12]. Hcy is thought to stimulate the generation of hydroxyl radicals, which leads to lipid peroxidation (LPO). Proteins and carbohydrates can be damaged by free radicals and LPO products such as 4-hydroxy-2-nonenal and malondialdehyde (MDA), a prominent end product of LPO [13,14]. Superoxide anions can also react quickly with nitric oxide (NO) to generate peroxynitrite, a highly reactive oxidant that can cause tissue damage [15,16]. Hcy can also decrease the expression of antioxidant enzymes like glutathione GSH peroxidase (GPx), which could help to eliminate the destructive effects of ROS [17]. The antioxidant proteins thioredoxin and heme oxygenase-1 (HO-1) are substantially downregulated by Hcy [18]. Several dietary components have been demonstrated to reduce the impact of HHcy, including folic acid and vitamins B6 and B12 [13]. In any case, health-care practitioners should create effective prevention and intervention strategies to combat this condition.

For many decades plants have been employed to heal human sicknesses. The cashew tree (*Anacardium occidentale* L.) is a Brazilian native that is now widely planted around the world. Cashew nuts, when consumed as part of a well-balanced diet, can help to reduce the risk of cardiovascular disease, particularly stroke, as well as metabolic syndrome [19]. An earlier study looked at the effects of dietary supplementation with industrial processing by-products like cashew (*Anacardium occidentale* L.) fruit on the intestinal health and lipid metabolism of rats with diet-induced dyslipidemia [20]. Nuts are regarded as an important part of a balanced diet, since they include protein, good fatty acids, and critical nutrients [21]. Natural antioxidants, such as polyphenol-rich meals, fresh fruits, and vegetables, may be able to counteract ROS oxidative degradation [22]. Unsaturated fatty acids (UFAs) such as oleic (-9) and linoleic (-6) acid, flavonoids, anthocyanins, tannins, fiber, folate, and tocopherols are abundant in cashew nuts [23]. The cashew nut and its derivatives have a wide range of biological capabilities, including antioxidant and antibacterial qualities [24]. Several animal studies have shown that cashew nuts have antioxidant and protective properties in the treatment of several inflammatory syndromes [25,26,27].

Based on these findings, and in particular considering the content of folate and flavonoids present in cashew nuts, the aim of this study was to investigate the anti-inflammatory and antioxidant effects of oral administration of cashew nuts in a rat model of HHcy induced by L-methionine oral injection.

## 2. Materials and Methods

### 2.1. Animals

Male Sprague Dawley rats (250 g, Envigo, Milan, Italy) were housed in a well-organized environment (room 22.1 °C, 12 h dark–light cycles) and fed normal rodent food and water. For one week, the animals were acclimatized to these conditions. The research was approved by the Animal Welfare Review Board at Messina University, protocol number: n° 897/2021-PR. All animal studies follow new Italian legislation (D.Lgs 2014/26), as well as EU Regulations (EU Directive 2010/63).

### 2.2. Cashew Nuts’ Nutritional Composition 

The cashew kernel samples (*Anacardium occidentale* L.) were obtained from the Ivory Coast; per 100 g, they contained 5.40 g moisture, 22.46 g protein, 44.19 g total lipids, 4.48 g total dietary fiber, 30.95 g total sugars, 2.68 g ash, and 80.01 mg total phenols. The nutritional composition was analyzed according to the Association of Official Analytical Chemists (AOAC) Official Method, as previously reported [25]. The total content of folate in cashew nuts is 25 µg/100 mg (https://fdc.nal.usda.gov/fdc-app.html#/food-details/170162/nutrients, accessed on 5 March 2022).

### 2.3. Animal Model Induction

Hyperhomocysteinemia was induced in male rats by methionine administration (meth) (1 g/kg, oral, 30 days) dissolved in drinking water [28]. 

### 2.4. Experimental Groups

The animals were randomly divided into groups and treated as follows:

Sham+vehicle: rats received only normal saline (instead of methionine) and were treated orally with saline for 30 days;

Sham+cashew nuts: rats received only normal saline (instead of methionine) and were treated orally with cashew nuts (100 mg/kg, oral) for 30 days;

Meth+vehicle: rats received methionine (1 g/kg, oral) for 30 days and were treated with saline;

Meth+cashew nuts: rats were subjected daily to methionine and received cashew nuts (100 mg/kg, oral) for 30 days.

Doses of cashew nuts were chosen based on previous studies [27].

Since no significant difference was found between the sham+vehicle and sham+cashew nuts groups, only data regarding the sham+vehicle groups were shown. At the end of experiment (30 days), the animals were sacrificed. Blood, colon, liver, and kidney tissues were collected from all groups. 

### 2.5. Biochemical Analyses

Serum levels of Hcy were assessed using a commercially available kit for HPLC measurements (Bio-Rad, Milan, Italy), according to the manufacturer’s instructions. The serum concentration of total cholesterol was assessed using a commercially available kit (Byosistems, Reagents and Instruments, Barcelona, Spain) by means of an automated analyzer UV spectrophotometer (model Slim SEAC, Florence, Italy). All sera were also analyzed for determination of the following parameters: aspartate transaminase AST, alanine aminotransferase ALT, lactate dehydrogenase LDH, and alkaline phosphatase ALP, using commercial kits (Abcam, Milan, Italy). The plasma creatinine concentrations were assayed as previously indicated [29,30].

### 2.6. Antioxidant Levels

The levels of superoxide dismutase SOD, glutathione GSH, and catalase CAT were assayed in the blood, according to the manufacturer’s instructions (Cusabio Biotech Co., Ltd., Wuhan, China) [23].

### 2.7. Malondialdehyde (MDA) Measurement 

Plasma malondialdehyde (MDA) levels were determined as an indicator of lipid peroxidation, as indicated [31]. A total of 100 μL of plasma was added to a mix of 200 μL of 8.1% SDS, 1500 μL of 20% acetic acid (pH 3.5), 1500 μL of 0.8% thiobarbituric acid, and 700 μL distilled water. Samples were then warmed for 1 h at 95 °C and centrifuged at 3000× *g* for 10 min. The absorbance was detected at 650 nm.

### 2.8. Cytokine Measurement

Plasma tumor necrosis factor alpha (TNF-α) and interleukin (IL-1β) were assessed using ELISA kits provided by R&D Systems, Minneapolis, MN, USA. 

### 2.9. Histological Examination 

For histological analysis, tissues were subjected to hematoxylin and eosin staining and observed by competent pathologists using a Leica DM6 microscope (Leica Microsystems SpA, Milan, Italy), associated with Leica LAS X Navigator software (Leica Microsystems SpA, Milan, Italy). Histological injuries were scored as previously reported [12,32,33]. Paraffin-embedded skin tissues with a thickness of 5 μm were stained with Masson’s trichrome, according to the manufacturer’s protocol (Bio-Optica, Milan, Italy) [34,35]. 

### 2.10. Immunohistochemical Localization of Poly (ADP-Ribose Polymerase) (PARP), Nitrotyrosine 

Immunohistochemical analysis was performed as previously described [36,37,38]. The sections were incubated overnight with primary antibodies: anti-PARP mouse polyclonal antibody (1:100 in PBS, *v*/*v*, Santa Cruz Biotechnology (SCB), and anti-nitrotyrosine rabbit polyclonal antibody (1:200 in PBS, *v*/*v*, Millipore). Sections were cleaned with PBS and then treated as indicated previously [36]. Five stained sections from each mouse were scored in a blinded fashion and observed using a Leica DM6 microscope (Leica Microsystems SpA, Milan, Italy) following a typical procedure [39]. The histogram profile was related to the positive pixel intensity value obtained [40].

### 2.11. Western Blots for Nuclear Factor NF-kB, NRF-2 and HO-1, and Bax and Bcl-2

Cytosolic and nuclear extracts were prepared as previously described [41]. The following primary antibodies were used: anti-NF-kB (SCB; 1:500 #sc8008), anti-NRF-2 (sc-365949, 1:1000, SCB), anti-HO-1 (sc-136960, 1:1000 SCB), anti-Bcl-2 (SCB, sc-7382), anti-Bax (SCB, sc-7480), in phosphate-buffered saline, 5% *w*/*v* non-fat dried milk, and 0.1% Tween-20 at 4 °C overnight. Membranes were incubated with peroxidase-conjugated bovine anti-mouse IgG secondary antibody or peroxidase-conjugated goat anti-rabbit IgG (Jackson ImmunoResearch, West Grove, PA, USA; 1:2000) for 1 h at room temperature. Anti-β-actin or anti-lamin A/C antibodies were used as controls. The expression of protein bands was detected by a procedure previously described [41]. To establish that the blots were loaded with identical volumes of lysate, they were also probed with anti-β-actin or anti-lamin A/C antibodies. Comparative expression of the protein bands was identified with a chemiluminescence detection procedure, following the manufacturer’s instructions (Super Signal West Pico Chemiluminescent Substrate; Pierce). The expression of protein bands was computed by densitometry with BIORAD ChemiDocTM XRS+software and standardized to β-actin or lamin A/C levels. Images of blot signals were imported to analysis software (Image Quant TL, v2003).

### 2.12. Terminal Deoxynucleotidyl Nick-End Labeling (TUNEL) Assay

Apoptosis was analyzed by a TUNEL assay using a cell death detection kit. TUNEL staining for apoptotic cell nuclei was performed as previously described [42]. 

### 2.13. Materials

All chemicals were analytical grade or higher. Methionine was purchased from Sigma Chemical (St. Louis, MO, USA).

### 2.14. Statistical Evaluation

All results are given as the mean standard error of the mean (SEM) of N observations. N = animal number. For histology/immunohistochemistry, at least three separate experiments resulted in the images. A *p* value of <0.05 was considered to indicate significance. For multiple comparisons, a one-way ANOVA was employed, followed by a Bonferroni post-hoc test.

## 3. Results

### 3.1. Effect of Cashew Nuts on Serum Hcy Levels after Methionine Administration

**Figure 1:**
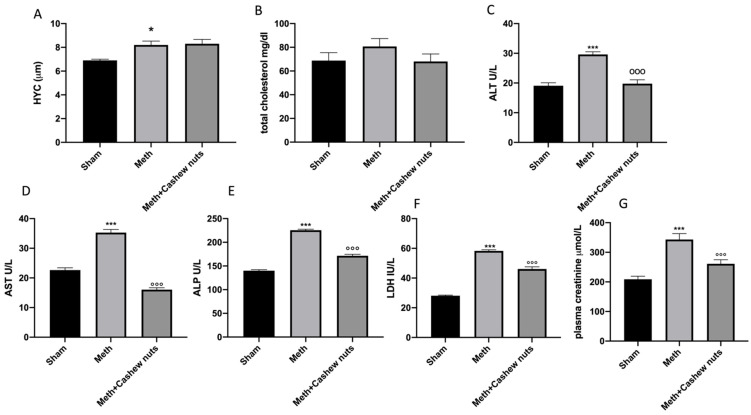
Evaluation of clinical biochemical parameters. Serum levels of Hcy (**A**); total cholesterol (**B**); ALT (**C**); AST (**D**); ALP (**E**); LDH (**F**); plasma creatinine (**G**). Values are means ± SEM of six animals for each group; * *p* < 0.05 vs. sham *** *p* < 0.001 vs. sham; °°° *p* < 0.001 vs. Meth.

In the present study, to see whether the oral administration of methionine effectively caused the condition HHcy, we measured the levels of Hcy in the serum. Methionine administration (1 g/kg oral) for 30 days caused an increased level of Hcy compared to the sham group (Figure 1A). However, the cashew nut treatment was not able to directly reduce the elevated serum levels of Hcy (Figure 1A).

Thus, the treatment with cashew nuts did not act directly on the reduction of serum Hcy levels, but may have reduced inflammation and oxidative stress due to the HHcy condition.

### 3.2. Effect of Cashew Nut Oral Administration on Biochemical Changes Induced by HHcy in Rats

To analyze the clinical effects of HHcy, we measured biomarkers to evaluate the lipidic profile (total cholesterol), and the functioning of the liver (ALT, AST, ALP, LDH) and kidneys (creatinine). An increase in serum total cholesterol, ALT, AST, ALP, and LDH concentrations was observed in L-methionine-induced HHcy rats compared to the sham group (Figure 1B–F). Cashew nut treatment caused a decrease in elevated serum total cholesterol, ALT, AST, ALP, and LDH concentrations when compared with the L-methionine administered group (Figure 1B–F). In addition, an increase in plasma creatinine was also observed in meth-subjected animals compared to the sham group (Figure 1G). Cashew nut treatment was able to reduce creatinine levels (Figure 1G). 

### 3.3. Effect of Cashew Nut Oral Administration on Oxidative Stress Induced by HHcy in Rats

**Figure 2:**
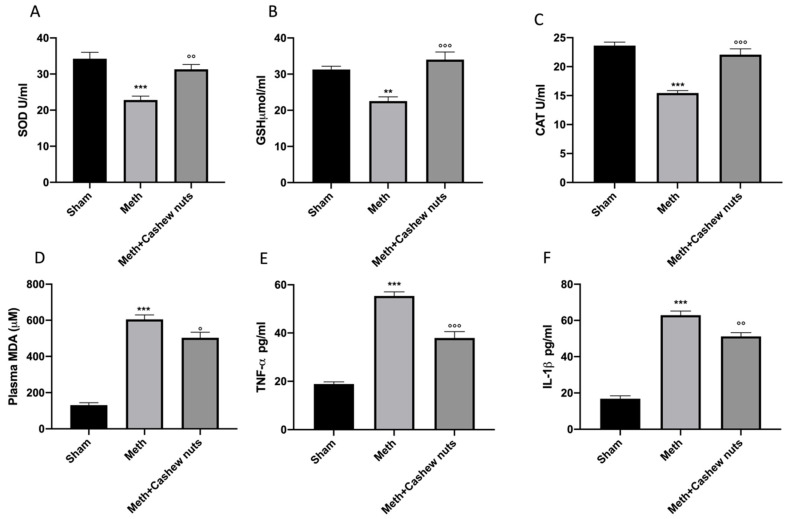
Evaluation of antioxidant, MDA, and cytokine levels. Serum levels of SOD (**A**); GSH (**B**); CAT (**C**); plasma MDA (**D**); TNF-α (**E**); IL-1β (**F**). Values are means ± SEM of six animals for each group; ** *p* < 0.01 vs. sham *** *p* < 0.001 vs. sham; °°° *p* < 0.001 vs. Meth. °° *p* < 0.01 vs. Meth. ° *p* < 0.05 vs. Meth.

A decrease in serum levels of SOD, CAT, and GSH was found in HHcy-subjected rats compared to the sham groups (Figure 2A–C). Oral administration of cashew nuts significantly increased all of the parameter levels (Figure 2A–C). In addition, an increase in MDA levels, an indicator of lipid peroxidation, was observed (Figure 2D). Cashew nuts reduced plasma MDA levels in a significant way (Figure 2D).

### 3.4. Effect of Cashew Nut Oral Administration on Cytokine Release Induced by HHcy in Rats

HHcy induced by oral methionine administration caused an increase in plasma cytokine release, specifically TNF-α and IL-1β, in HHcy vehicle rats compared to the controls (Figure 2E,F). Cashew nut treatment was able to decrease pro-inflammatory cytokine release in a significant way (Figure 2E,F).

### 3.5. Effect of Cashew Nut Oral Administration on Histological Damage and Fibrosis Induced by HHcy in Rats

**Figure 3:**
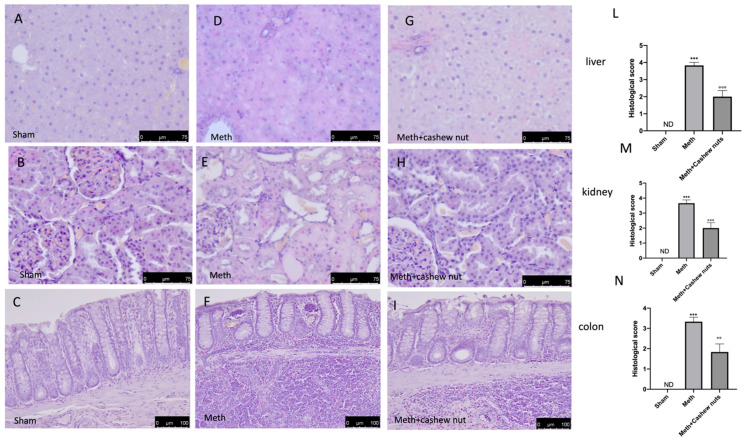
The effects of cashew nuts on histological damage in HHcy rats. Histological analysis was evaluated in the sham (**A**–**C**); Meth (**D**–**F**); and Meth+cashew nuts (**G**–**I**) groups, respectively, in the liver, kidney, and colon sections. Histological scores are shown (**L**–**N**). Figures are representative of at least three independent experiments. Values are means ± SEM of six animals for each group. *** *p* < 0.001 vs. sham; °°° *p* < 0.001 vs. Meth. °° *p* < 0.01 vs. Meth., ND not detectable. Scale bar 100 μm and 75 μm. Magnification (20X and 40X).

**Figure 4:**
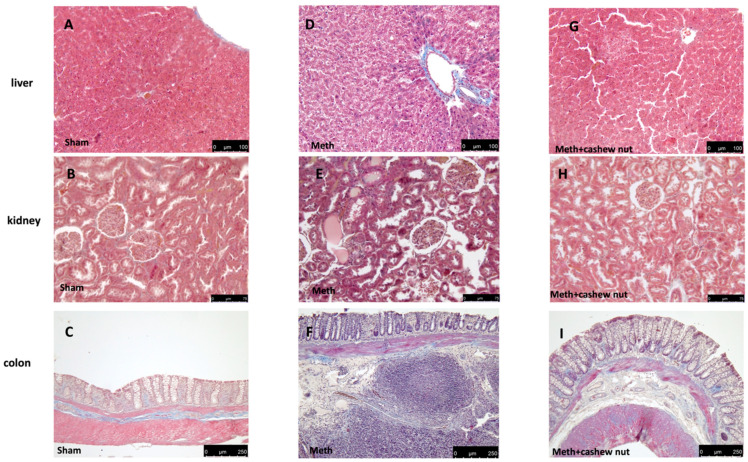
The effects of cashew nuts on fibrosis in HHcy rats. Masson’s trichrome staining was performed in the sham (**A**–**C**); Meth (**D**–**F**); and Meth+cashew nuts (**G**–**I**) groups, respectively, in the liver, kidney, and colon sections. The connective tissue is dyed blue, the cytoplasm is colored red/pink, and the nuclei are stained dark red/purple. The figures are representative of at least three independent experiments. Scale bar: 250 μm, 100 μm, and 75 μm. Magnification (10X; 20X; and 40X).

Oral methionine administration caused an important histological alteration in the kidney, colon, and liver tissues, with necrosis, inflammation, and cellular infiltrate observed (Figure 3D–F, and see scores Figure 3L–N) compared to the sham group (Figure 3A–C, and see scores Figure 3L–N). Cashew nut treatment significantly reduced the histological injury in all tissues (Figure 3G–I, and see scores Figure 3L–N). Additionally, Masson’s trichrome was performed to evaluate the fibrotic process by deposition of collagen in the liver, colon, and kidney tissues. This stain showed an increase of collagen formation in HHcy rats treated with the vehicle (Figure 4D–F) compared to the sham group (Figure 4A–C). Cashew nuts reduced collagen formation in all tissues (Figure 4G–I).

### 3.6. Effect of Cashew Nut Oral Administration on Nitrotyrosine and PARP in HHcy Rats

**Figure 5:**
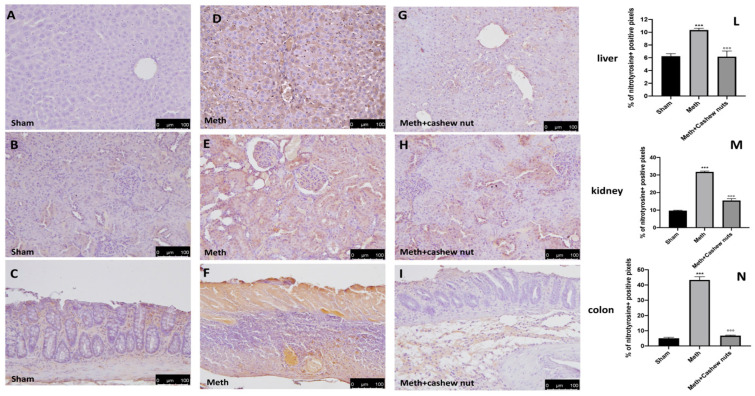
The effects of cashew nuts on nitrotyrosine expression in HHcy rats. Immunohistochemistry for nitrotyrosine was evaluated in the sham (**A**–**C**); Meth (**D**–**F**); and Meth+cashew nuts (**G**–**I**) group in the liver, kidney, and colon sections, respectively. The results are expressed as the percentage of positive pixels (**L**–**N**). The figures are representative of at least three independent experiments. Values are the means ± SEM of six animals for each group; *** *p* < 0.001 vs. sham, °°° *p* < 0.001 vs. Meth. Scale bar: 100 μm. Magnification 20X.

**Figure 6:**
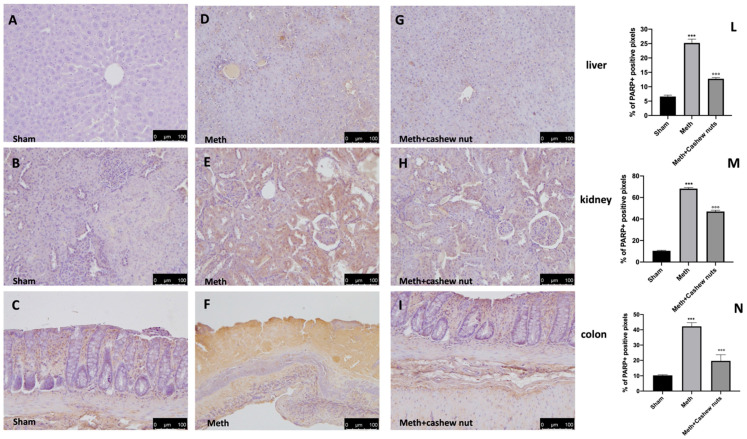
The effects of cashew nuts on PARP expression in HHcy rats. Immunohistochemistry for PARP was evaluated in the sham (**A**–**C**); Meth (**D**–**F**); and Meth+cashew nuts (**G**–**I**) groups, respectively, in the liver, kidney, and colon sections. The results are expressed as the percentage of positive pixels (**L**–**N**). The figures are representative of at least three independent experiments. Values are means ± SEM of six animals for each group; *** *p* < 0.001 vs. sham, °°° *p* < 0.001 vs. Meth. Scale bar: 100 μm. Magnification 20X.

The expression of nitrotyrosine, a specific indicator of nitrosative stress, and PARP, an indicator of DNA breakdown, was analyzed by immunohistochemical staining. Sections of colon, liver, and kidney tissues from the sham rats did not show marks for nitrotyrosine (Figure 5A–C and see Figure 5L–N), whereas sections from the HHcy rats demonstrated a robust positive staining for nitrotyrosine (Figure 5D–F, and see Figure 5L–N). In addition, increased PARP-positive staining was also observed in tissues from the HHcy rats (Figure 6D–F, and see Figure 6L–N) compared to the sham group (Figure 6A–C and see Figure 6L–N). Oral treatment with cashew nuts at 100 mg/kg significantly reduced positive staining for nitrotyrosine and PARP in all tissues (Figure 5G–I and Figure 6G–I, and see Figure 5L–N and Figure 6L–N). 

### 3.7. Effect of Cashew Nut Oral Administration on NF-kB, NRF-2, and HO-1 Expression in HHcy Rats

**Figure 7:**
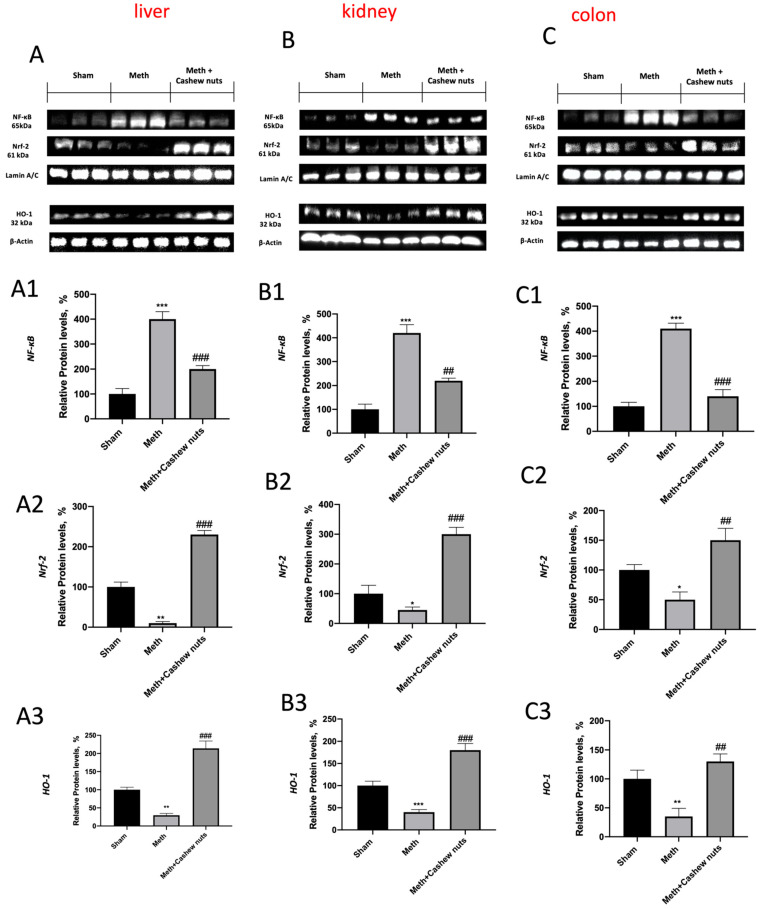
The effects of cashew nuts on the NRF-2 and NF-kB pathways in HHcy rats. Representative Western blots for NF-κB, NRF-2, and HO-1 ((**A**–**C**) for the liver, kidney, and colon tissues) were performed. Shown is a representative blot of lysates from six animals per group, together with a densitometric analysis normalized to housekeeping proteins. The results in (**A1**–**A3**,**B1**–**B3**,**C1**–**C3**) are expressed as the relative protein level percentage, and means ± SEM, of six animals for each group. *** *p* < 0.001 vs. sham; ** *p* < 0.01 vs. sham, * *p* < 0.05 vs. sham, ^###^ *p* < 0.001 vs. Meth. ^##^ *p* < 0.01 vs. Meth.

To better investigate whether, in HHcy, cashew nuts may act by interacting with signaling pathways such as nuclear NF-kB or Nrf-2/ HO-1, Western blots for the NF-kB and NRF-2/HO-1 pathways were also performed with liver, kidney, and colon tissues. Increased nuclear NF-kB and reduced Nrf-2 expression were observed in response to HHcy intervention with respect to the sham animals (Figure 7A,A1,A2,B,B1,B2,C,C1,C2). Cashew nuts significantly reduced the level of nuclear NF-kB, as well as upregulating Nrf-2 compared with the HHcy vehicle group, suggesting that cashew nuts diminish nuclear translocation of NF-kB and increase Nrf-2 (Figure 7A,A1,A2,B,B1,B2,C,C1,C2). At the same time, Western blot analysis showed that cashew nut treatment significantly enhanced the HHcy-induced decrease in HO-1 protein expression (Figure 7A,A3,B,B3,C,C3).

### 3.8. Effect of Cashew Nut Oral Administration on Apoptosis in HHcy Rats

**Figure 8:**
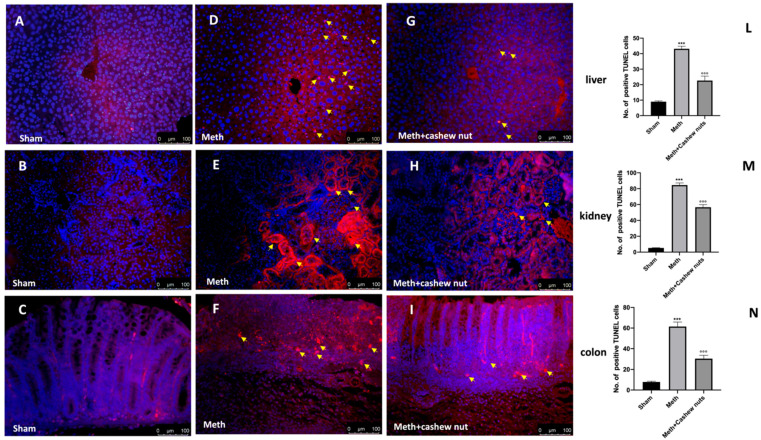
The effects of cashew nuts on apoptosis according to TUNEL assay in HHcy rats. The presence of apoptotic fragments was evaluated by TUNEL assay in the sham (**A**–**C**); Meth (**D**–**F**); and Meth+cashew nuts (**G**–**I**) groups, respectively, in the liver, kidney, and colon sections. The number of TUNEL-positive cells (yellow arrows) was counted in three sections per animal, and is presented as the number of positive cells per high-power field (**L**–**N**). For TUNEL staining, 100 μm scale bar. Magnification 20X. Figures are representative of at least three independent experiments. Values are means ± SEM of six animals for each group; *** *p* < 0.001 vs. sham, °°° *p* < 0.001 vs. Meth.

**Figure 9:**
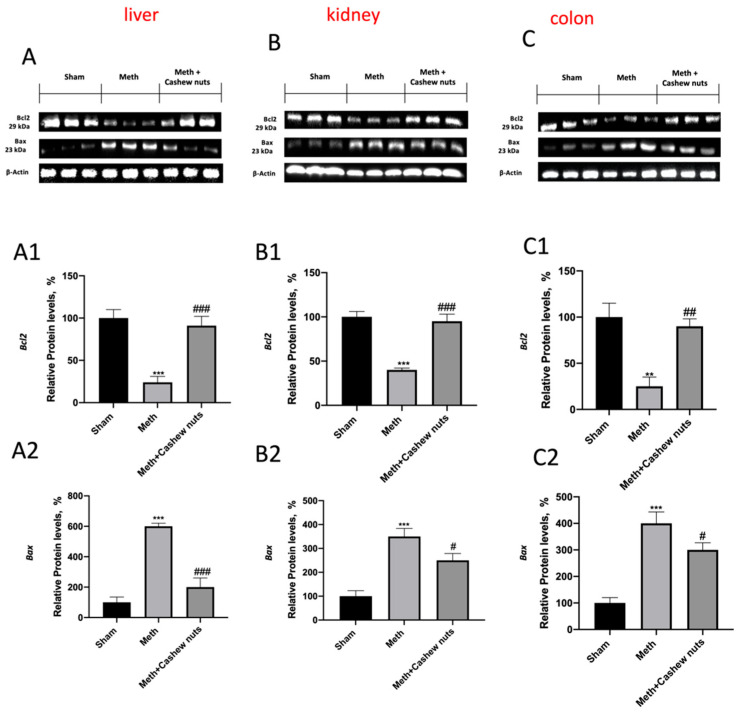
The effects of cashew nuts on apoptosis by Western blot for Bax and Bcl-2 in HHcy rats. Representative Western blots for Bax and Bcl-2 ((**A**–**C**) for liver, kidney, and colon tissues) were performed. Shown is a representative blot of lysates from six animals per group, together with a densitometric analysis normalized to housekeeping proteins. The results in (**A1**,**A2**,**B1**,**B2**,**C1**,**C2**) are expressed as relative protein level percentages and means ± SEM of six animals for each group. *** *p* < 0.001 vs. sham; ** *p* < 0.01 vs. sham, ^###^ *p* < 0.001 vs. Meth. ^##^ *p* <0.01 vs. Meth. ^#^ *p* < 0.05 vs. Meth.

In order to assess whether the damage of HHcy induced by methionine was also associated with apoptosis, we performed a TUNEL assay and Western blot analyses. In the liver, kidney, and colon sections, the TUNEL assay was utilized to determine how many cells were experiencing apoptosis. A low level of TUNEL-positive staining was detected in the sham group (Figure 8A–C, and see Figure 8L–N), whereas a significantly increased number of TUNEL-positive cells were observed in the HHcy rats (Figure 8D–F, and see Figure 8L–N). Administration of cashew nuts reduced the number of TUNEL-positive cells (Figure 8G–I, and see Figure 8L–N). Methionine administration also significantly increased the expression of Bax (pro-apoptotic) and decreased that of Bcl-2 (anti-apoptotic) (Figure 9A,A1,A2,B,B1,B2,C,C1,C2). Cashew nut treatment significantly downregulated HHcy-induced Bax expression and significantly increased the levels of Bcl-2, exerting a significant anti-apoptotic effect (Figure 9A,A1,A2,B,B1,B2,C,C1,C2).

## 4. Discussion

HHcy is a methionine metabolism abnormality that can cause a variety of disorders in humans, including cardiovascular and neurological conditions like atherosclerosis and stroke; inflammatory syndromes, including osteoporosis and rheumatism; and Alzheimer’s and Parkinson’s diseases. Meth’s biochemistry is tightly controlled by a number of enzymes that regulate Hcy levels. Certainly, the cell’s well-being depends on balanced enzyme activity, and its failure might result in an increase in the Hcy concentration, which could contribute to the start of a variety of pathological disorders. HHcy may be a disorder involving the dysfunction of more organs, such as the kidney, liver, or gut, which are currently poorly understood, putting a greater emphasis on the need to invest in research. HHcy caused by dietary folate restriction promotes oxidative stress in the kidneys or generates ROS release, inflammatory infiltration, and fibrosis, and lowers the glycogen/glycoprotein concentration in the liver of rats [1,43]. Several reports have also revealed that high blood Hcy levels are a major risk factor for chronic kidney disease [44]. A calibrated assumption of correct vitamin doses, such as folate, vitamin B6, vitamin B12, and betaine, may be effective in controlling HHcy-related diseases [45,46]; as a result, daily consumption of these micronutrients must be examined. Because nuts include proteins, healthy fatty acids, and critical elements, a daily balanced consumption of nuts is important for good health. The oxidative effect of ROS could be neutralized by eating polyphenol-rich foods, fresh fruits, and vegetables. The cashew nut is one of the four most well-known nuts in the world, thanks to its high nutritional value and unique flavor. Several of our studies have demonstrated the beneficial effects of cashew nuts in different inflammatory experimental models, in particular in colitis, pancreatitis, ischemia, and osteoarthritis [23,25,26,27,47]. Studying the status of HHcy in animal models could help researchers to better understand the mechanisms underlying the disease, and create effective preventative and intervention strategies for HHcy-induced tissue alterations [48]. L-methionine-treated rats are typically used to examine HHcy and its downstream consequences. Because high concentrations of Hcy can harm cells and play a role in the production and progression of tissue damage, substances that can reduce oxidative stress may be useful in this process; cashew nuts may be one food with this ability.

Based on this, and considering the content of folate and flavonoids present, the aim of this work was to evaluate the anti-inflammatory and antioxidant effects of cashew nuts in HHcy and examine the possible pathways involved. In HHcy rats, oral administration of cashew nuts (100 mg/kg) was able to counteract clinical biochemical changes, oxidative and nitrosative stress, reduced antioxidant enzyme levels, lipid peroxidation, proinflammatory cytokine release, and histological tissue injuries, fibrosis, and apoptosis, respectively, in the kidney, colon, and liver. Our findings are consistent with prior research that found methionine supplementation increased plasma Hcy levels, induced oxidative stress, and decreased antioxidative enzyme activity [49]. HHcy has been shown to cause mitochondrial dysfunction through regulating oxidative stress [50,51], and may promote the generation of H_2_O_2_ and hydroxyl radicals via the autoxidation of sulfhydryl (-SH) groups or by decreasing the intracellular levels of GSH, which is implicated in the abolition of free radicals [52]. Hcy-induced reductions in Nrf2 expression, as well as reduced antioxidant enzyme expression/activity and increased ROS generation, have been widely reported [51]. In response to various types of stimulation, such as oxidative stress, Nrf2 is the major transcriptional activator of the HO-1 gene [53]. The Nrf2/HO-1 pathway can protect cells from oxidative stress-induced damage when it is activated [54]. Antioxidants are strong activators of Nrf2 because, after metabolism, they generate a minor quantity of oxidative stress that supports Nrf2 activation [55,56]. High levels of Hcy significantly repress HO-1 mRNA and protein expression in HepG2 cells [49]. Inhibition of SOD activity is also one of the mechanisms of Hcy-induced oxidative stress [13]. Derouiche et al. discovered that Hcy inhibits SOD and CAT activities in rats [57]. In the present study, we found that cashew nut treatment was able to promote Nrf2 nuclear translocation and to induce the expression of Nrf2, and regulated factors such as HO-1 in all tissues, along with increasing the serum levels of SOD, GSH, and CAT. This is in agreement with previous studies in which cashew nuts were able to activate the NRF2/HO-1 pathway [26]. 

It has also been widely reported that HHcy induces acute and chronic inflammatory events via NF-κB regulation [12]. In a neuroblastoma cell line, an induced high level of Hcy was demonstrated to boost NF-kB levels, and this was inhibited by the introduction of antioxidants [58]. HHcy was also reported to promote the production of IL-1β and TNF-α by human peripheral blood monocytes [59,60]. A previous study reported that in inflamed lungs, animals given anacardic acids from cashew nuts had lower levels of neutrophils and TNF, respectively [61]. In addition, cashew nut administration reduced the levels of cytokines in other animal models [23,25,26,27,47]. The release of proinflammatory cytokines is regulated by intracellular signal transduction, such as the NF-κB pathway. In this study, we demonstrated that cashew nuts cause a reduction of NF-κB expression, as well as pro-inflammatory TNF-α and IL-1β levels. Peroxynitrite is a highly reactive oxidant that damages cells by altering lipids, proteins, and DNA. A considerable increase in the amounts of lipid peroxides and nitrotyrosine protein adducts in hyperhomocysteinemic rats was previously observed [62. Based on these findings, in this study we demonstrated that oral treatment with cashew nuts was able to significantly reduce lipid peroxidation by altering plasma MDA levels, nitrotyrosine production, and PARP activation in all tissues.

The link between ROS and apoptosis is well known. Previous research has found that excessive Hcy levels cause cardiomyocyte apoptosis or necrosis by increasing oxidant stress [63]. Furthermore, Hcy administration increases the levels of many pro-apoptotic markers, such Bax, p53, and caspase-3, implying an association between HHcy-induced cell damage and NF-kB activation [58]. Here, we also demonstrated an increased expression of proapoptotic protein Bax and decreased expression of antiapoptotic Bcl-2 by Western blot analysis, as well as an augmented presence of apoptotic fragments by TUNEL assay detection in all tissues in HHcy rats. Cashew nut treatment was able to reduce the apoptotic process.

In conclusion, cashew nuts were able to ameliorate tissue inflammation and oxidative stress, possibly through the regulation of ROS-induced signaling, such as nuclear NRF-2 or NF-κB, and increased antioxidant capacity. Thus, the balanced consumption of cashew nuts could be beneficial for inflammatory events associated with HHcy.
