# Correction: D’Amico et al. Consumption of Cashew (*Anacardium occidentale* L.) Nuts Counteracts Oxidative Stress and Tissue Inflammation in Mild Hyperhomocysteinemia in Rats. *Nutrients* 2022, *14*, 1474

**DOI:** 10.3390/nu16010133

**Published:** 2023-12-30

**Authors:** Ramona D’Amico, Marika Cordaro, Roberta Fusco, Alessio Filippo Peritore, Tiziana Genovese, Enrico Gugliandolo, Rosalia Crupi, Giuseppina Mandalari, Daniela Caccamo, Salvatore Cuzzocrea, Rosanna Di Paola, Rosalba Siracusa, Daniela Impellizzeri

**Affiliations:** 1 Department of Chemical, Biological, Pharmaceutical and Environmental Sciences, University of Messina, Via F. Stagno D’Alcontres 31, 98166 Messina, Italy; rdamico@unime.it (R.D.); aperitore@unime.it (A.F.P.); tgenovese@unime.it (T.G.); gmandalari@unime.it (G.M.); rsiracusa@unime.it (R.S.); dimpellizzeri@unime.it (D.I.); 2 Department of Biomedical, Dental and Morphological and Functional Imaging, University of Messina, Via Consolare Valeria, 98125 Messina, Italy; cordarom@unime.it (M.C.); daniela.caccamo@unime.it (D.C.); 3 Department of Clinical and Experimental Medicine, University of Messina, 98125 Messina, Italy; 4 Department of Veterinary Sciences, University of Messina, 98168 Messina, Italy; egugliandolo@unime.it (E.G.); rcrupi@unime.it (R.C.); 5 Department of Pharmacological and Physiological Science, Saint Louis University School of Medicine, 1402 South Grand Blvd, St. Louis, MO 63104, USA

In the original publication [1], there was a misunderstanding in Figure 5 as published. The authors incubated two different antibodies on two consecutive sections from the same sample (see Figure 5 panel D and Figure 6 panel D). In the correction, Figure 5 panel D has been replaced. The corrected Figure 5 appears below. The authors apologize for any inconvenience caused and state that the scientific conclusions are unaffected. This correction was approved by the academic editor. The original publication has also been updated.

**Figure 5:**
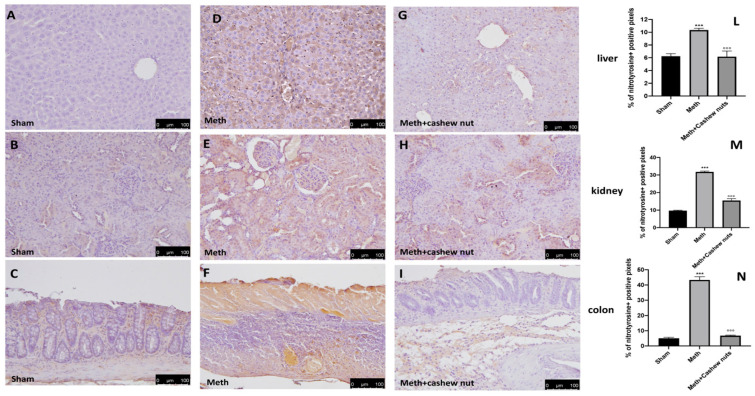
The effects of cashew nuts on nitrotyrosine expression in HHcy rats. Immunohistochemistry for nitrotyrosine was evaluated in the sham (**A**–**C**); Meth (**D**–**F**); and Meth+cashew nuts (**G**–**I**) group in the liver, kidney, and colon sections, respectively. The results are expressed as the percentage of positive pixels (**L**–**N**). The figures are representative of at least three independent experiments. Values are the means ± SEM of six animals for each group; *** *p* < 0.001 vs. sham, °°° *p* < 0.001 vs. Meth. Scale bar: 100 μm. Magnification 20X.
